# Promoter hypomethylated PDZK1 acts as a tumorigenic gene in glioma by interacting with AKT1

**DOI:** 10.18632/aging.205750

**Published:** 2024-04-25

**Authors:** Xing Ren, Dan Deng, Shasha Xiang, Jianbo Feng

**Affiliations:** 1 Clinical Laboratory Medicine Center, The First Affiliated Hospital of University of South China, Hengyang 421001, Hunan, P.R. China; 2 Cancer Research Institute, The First Affiliated Hospital, Hengyang Medical School, University of South China, Hengyang 421001, Hunan, P.R. China

**Keywords:** hypomethylation, PDZK1, glioma, AKT1

## Abstract

Glioma is the most frequently diagnosed primary brain tumor and typically has a poor prognosis because of malignant proliferation and invasion. It is urgent to elucidate the mechanisms driving glioma tumorigenesis and develop novel treatments to address this deadly disease. Here, we first revealed that PDZK1 is expressed at high levels in gliomas. Promoter hypomethylation may cause high expression of PDZK1 in glioma. Knockdown of PDZK1 inhibits glioma cell proliferation and invasion *in vitro*. Mechanistically, further investigations revealed that the loss of PDZK1 expression by siRNA inhibited the activation of the AKT/mTOR signaling pathway, leading to cell cycle arrest and apoptosis. Clinically, high expression of PDZK1 predicts a poorer prognosis for glioma patients than low expression of PDZK1. Overall, our study revealed that PDZK1 acts as a novel oncogene in glioma by binding to AKT1 and maintaining the activation of the AKT/mTOR signaling pathway. Thus, PDZK1 may be a potential therapeutic target for glioma.

## INTRODUCTION

Glioma is the most common type of primary brain tumor, and the incidence of gliomas has increased to the tenth highest among cancers in China. However, the etiology of this disease remains largely unknown. Gliomas typically escape microscopic surgical resection and reoccur because of their ability to diffusely invade the brain parenchyma [1]. Traditional surgical treatment cannot significantly increase the survival rate of glioma patients. Therefore, there is a great need to elucidate the mechanisms driving glioma tumorigenesis and develop novel treatments to address this deadly disease.

The PI3K/AKT signaling pathway is one of the important pathways regulating normal and cancer growth [2]. AKT is phosphorylated and activated by PI3K, which is anchored in the cell membrane [3]. The kinase activity of AKT is also regulated by other kinases, such as PDK1, which directly phosphorylates Thr308 of AKT, and mTORC2, which phosphorylates Ser473 of AKT [4]. AKT regulates the phosphorylation of several downstream proteins, such as CREB [5], p27KIP1 [6], Forkhead box O-class (FOXO) transcription factors [7], and mTOR, in a direct or indirect manner by promoting cell survival and growth [8]. The AKT signaling pathway is overactive in most cancer types, and activation of AKT protects cells against apoptosis and accelerates tumor cell growth. For example, mutations in PIK3CA and PTEN are frequently observed in several cancer types, and these mutations lead to activation of the PI3K/AKT/mTOR pathway, thus promoting cell survival and growth [9–11]. AKT signaling also functions as an oncogenic factor in gliomagenesis. AKT is activated in up to 90% of all glioblastomas [12], and targeting AKT signaling could be a useful therapeutic strategy for glioma [13].

PDZK1, a member of the PDZ domain, was first identified in human carcinomas [14]. The name PDZK1 is based on the presence of four domains that bind with the PDZ protein in its sequence. Most scientific studies have suggested that PDZK1 directly interacts with the C-terminus of HDL and SR-B1 through the domains that bind with the PDZ protein [15, 16]. Thus, PDZK1 plays an important role in HDL signaling regulated by scavenger receptor class B type I vascular cells. Furthermore, in recent renal cell carcinoma studies, PDZK1 was found to inhibit tumor development and progression [17, 18]. These studies supported that PDZK1 functions as a tumor suppressor in renal cell carcinoma (RCC) and provided original insight into the function and role of PDZK1 in tumorigenesis.

In our study, differential gene expression analysis using the CGGA and TCGA datasets revealed that PDZK1 was highly expressed in glioma tissues and that the PDZK1 expression level was correlated with tumor histologic grade. We first found that the expression of PDZK1 was correlated with adverse outcomes in glioma patients. Knocking down PDZK1 led to cell cycle arrest and cell apoptosis by inhibiting the AKT/mTOR signaling pathway in glioma cells. Our data indicated that PDZK1 may act as a cancer-promoting gene in glioma and may be an underlying therapeutic target for glioma.

## MATERIALS AND METHODS

### Data mining and analysis

PDZK1 and AKT1 gene expression, clinical correlation and prognosis data were obtained from the GEPIA and CGGA databases and then analyzed via the GEPIA (http://gepia.cancer-pku.cn/) and CGGA (http://www.cgga.org.cn/) websites. The Human Protein Atlas (http://www.proteinatlas.org/) dataset was used to analyze the expression level of PDZK1 in the normal cerebral cortex and glioma tissues. The methylation levels of PDZK1 CpG islands were analyzed by utilizing the EWAS Data Hub (https://ngdc.cncb.ac.cn/ewas/datahub/) in this study. Cancer genomes and clinical data were analyzed. The genomic map of PDZK1 was generated by using the cBioPortal platform (https://www.cbioportal.org/) in this study. The UALCAN database (http://ualcan.path.uab.edu/) was utilized to investigate the changes in DNA methylation of PDZK1 in glioma.

### Cell culture

The glioma cell lines U251 and U87 were purchased from Cell Banks. The U251 cells were cultured in DMEM (HyClone, USA), and the U87 cells were cultured in MEM (HyClone, USA) supplemented with 10% FBS (GIBCO, USA), 100 U/ml penicillin and 100 μg/ml streptomycin (HyClone, USA) at 37° C in a humidified incubator. The RNA interference (RNAi) sequence used for PDZK1 was 5’-CAAAGAAACUGACAAGCGUdT-3’, and the RT-qPCR primers used for PDZK1 were F- 5’-TTCCTGCGAATTGAGAAGGAC-3’ and R-5’-TCCACCCGTGTTTT CACTGC-3’.

### Transwell invasion assays

Cell invasion was measured by Transwell invasion assays. Briefly, U251/U87 cells (3x10^4 cells) were suspended in 1% FBS-containing growth medium over a Matrigel coating, and the lower chamber was cultured with 10% FBS-containing growth medium as a chemoattractant. The cell culture medium was supplemented with hydroxyurea to inhibit cell growth. Invaded cells were stained with 4 mg/ml calcein AM dye and then counted under a light microscope.

### CCK-8 cell proliferation assay

The protocols were described in our previous reports [19].

### EdU staining assay

DNA replication in glioma cells was determined using an EdU kit according to the manufacturer’s protocol. Briefly, 5x10^3 cells in 150 μl of medium were plated on a 96-well plate, and after being cultured for 4 h, the medium was replaced with 10 μM EdU medium and incubated for another 12 h. Apollo staining and nuclear staining were subsequently performed, after which the cells were counted using a fluorescence microscope.

### Flow cytometry analysis

For cell cycle analysis, cells were collected and fixed in ice-cold 70% ethanol at 4° C overnight, followed by washing with PBS and incubation with PI staining solution containing PI and RNase A. At least 2x10^4 cells were analyzed with a FACSCalibur flow cytometer.

### Western blot analysis

The protocols were described in our previous reports [19]. The primary antibodies used were as follows: anti-FLAG, anti-AKT1, anti-p-AKT1, anti-mTOR, anti-p-mTOR (CST), and anti-GAPDH and anti-PDZK1 (Proteintech, USA).

### Coimmunoprecipitation

The protocols were described in our previous reports [19].

### Colony formation assay

Glioma cells transfected with PDZK1 or scrambled siRNA were cultured for 24 hours. Then, the cells were digested, and 1000 cells from each group were inoculated into a culture dish. Cell culture was performed for 14 days and then terminated. After washing with PBS, the cells were fixed with 4% paraformaldehyde for 30 min. The cells were stained with 4 mg/ml calcein AM dye and then counted under a light microscope.

### Statistical analysis

The experiments were performed at least three times. The data are expressed as the mean ± standard deviation (SD). Statistical analysis was performed using GraphPad Prism 9 software. Student’s t-tests were used to determine significant differences between two groups. p < 0.05 was considered to indicate statistical significance.

## RESULTS

### PDZK1 is overexpressed in glioma and is correlated with poor prognosis

**Figure 1:**
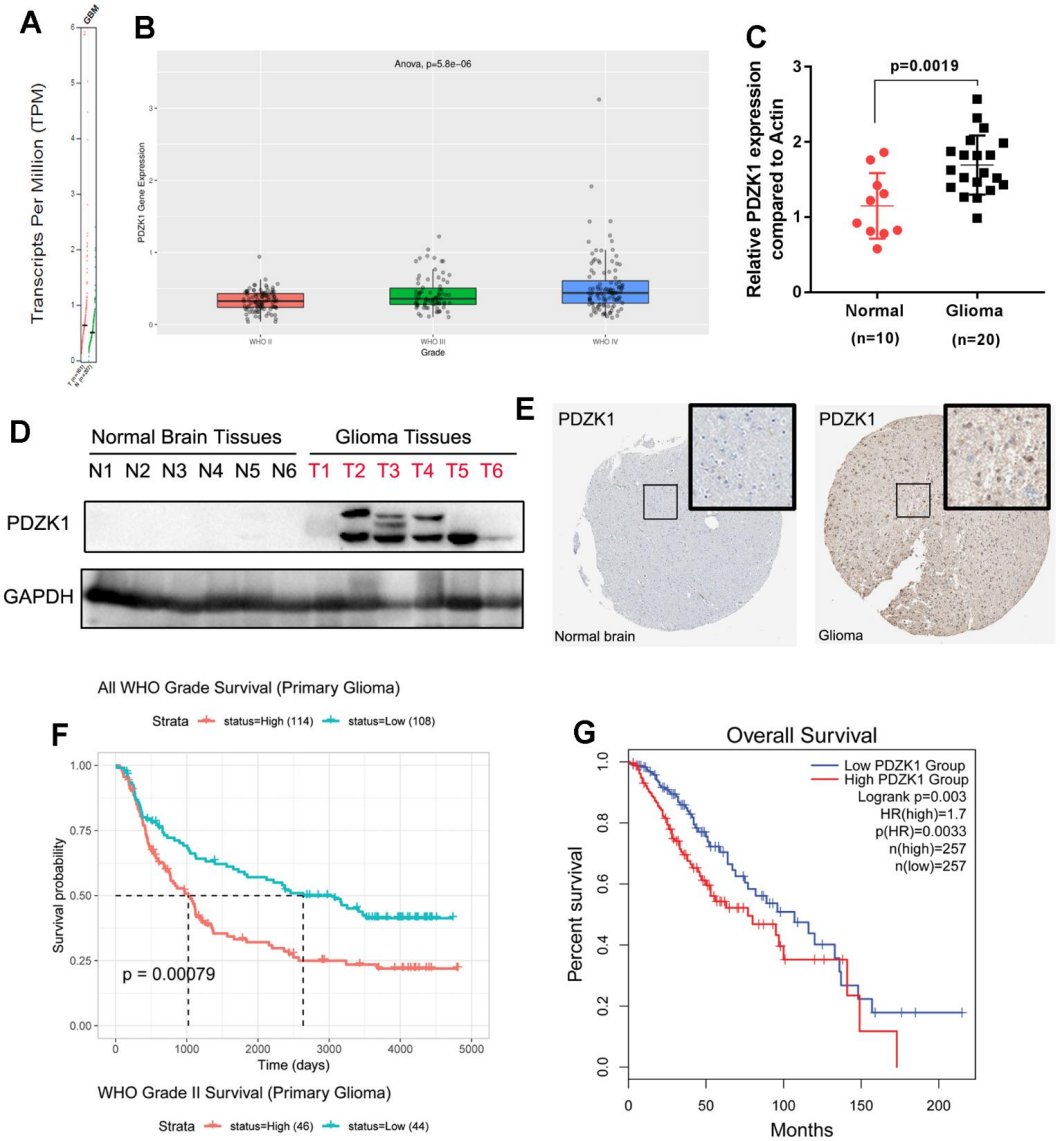
**PDZK1 is upregulated in glioma tissues, and high PDZK1 expression is associated with poor prognosis.** (**A**) The PDZK1 expression data in glioma tissues were obtained from TCGA datasets (GEPIA); GBM tissues have a higher PDZK1 expression level than normal tissues. (**B**) PDZK1 expression data were obtained from CGGA datasets; the PDZK1 expression level was associated with tumor histologic grade. (**C**) Real-time qPCR was used to detect the mRNA expression of PDZK1 in glioma tissues, and the expression of PDZK1 was much greater in glioma tissues than in normal brain tissues (p=0.0019). (**D**) Western blotting was used to detect the protein expression of PDZK1 in glioma tissues, and the protein expression of PDZK1 was much greater in glioma tissues than in normal brain tissues. (**E**) Immunohistochemical staining showed that PDZK1 expression is much greater in glioma tissues than in normal brain tissues, as shown in the image obtained from The Human Protein Atlas. (**F**, **G**) Glioma patients with lower PDZK1 expression had a more favorable survival time than did glioma patients with higher PDZK1 expression. The Kaplan–Meier method was used for this analysis. (**F**) data from CGGA; (**G**) data from TCGA.

By analyzing the TCGA dataset, we found that PDZK1 is present at higher levels in GBM tissues than in normal controls (Figure 1A). Furthermore, by using the CGGA database (mRNAseq_325), we also found that the expression level of PDZK1 was correlated with glioma histologic grade (Figure 1B). Next, to verify the expression of PDZK1 in glioma, 10 normal brain tissues and 20 glioma tissues were obtained for real-time fluorogenic quantitative PCR. The results also confirmed that PDZK1 was dramatically overexpressed in glioma specimens (Figure 1C), which is in accordance with results from TCGA and CGGA data mining and analysis.

What is the level of PDZK1 protein expression between normal and glioma tissues? Next, we examined PDZK1 protein expression in normal brain tissues and glioma tissues. The PDZK1 protein was highly expressed in most glioma tissues but was not expressed in all normal brain specimens. The HUMAN PROTEIN ATLAS dataset was also utilized to analyze the protein expression level of PDZK1 in the cerebral cortex and glioma. We also found that PDZK1 presented relatively high staining intensities compared to those in the cerebral cortex (Figure 1E). Moreover, survival curve analysis revealed longer survival times for glioma patients with relatively lower PDZK1 expression than for those with high PDZK1 expression in the CGGA (mRNAseq_325), TCGA (GEPIA:LGG) and GSE30074 datasets (Figure 1F, 1G and Supplementary Figure 1). Overall, our results indicated that PDZK1 may play a cancer-promoting role in glioma and that PDZK1 expression may be a prognostic factor in glioma.

### Promoter hypomethylation causes high expression of PDZK1 in glioma

**Figure 2:**
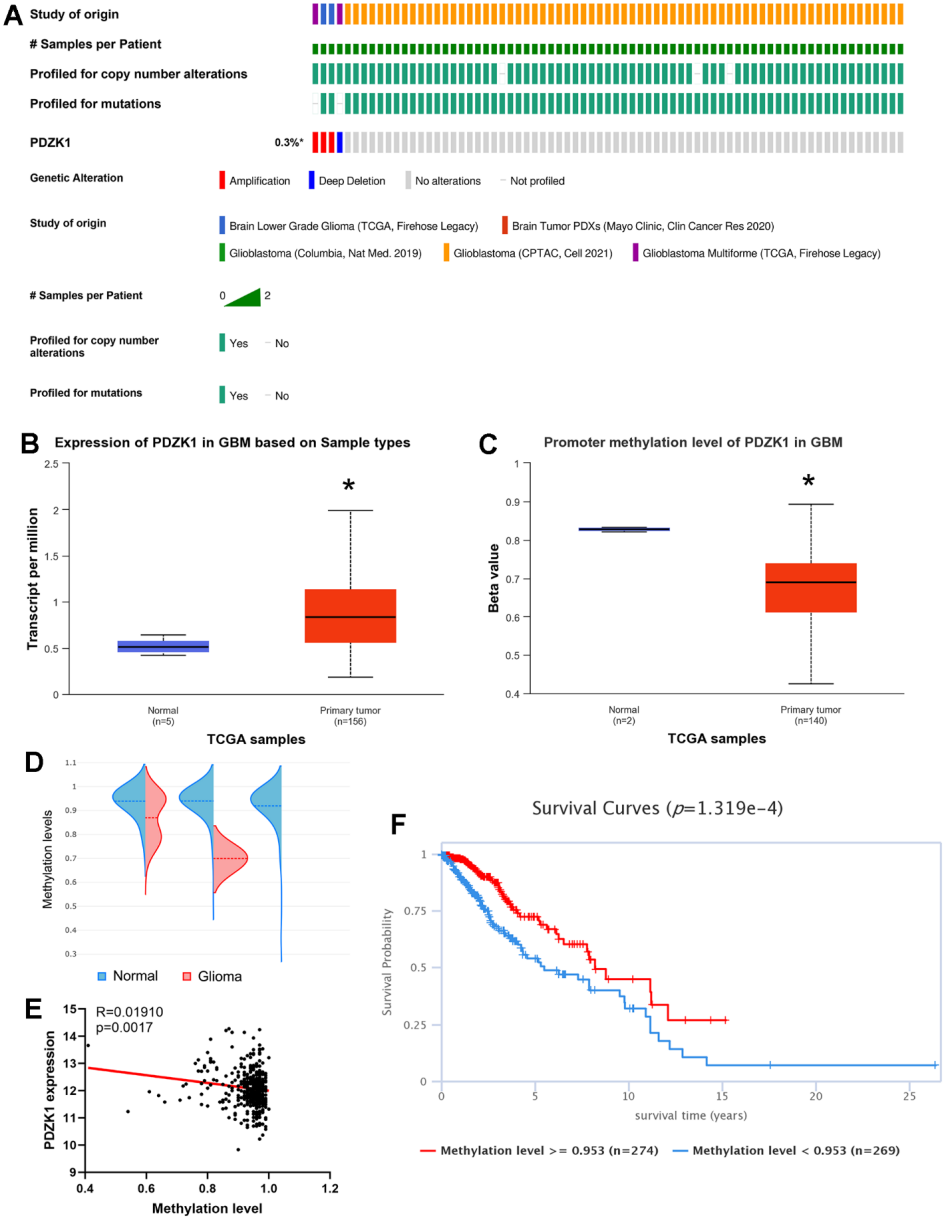
**Genetic alterations and DNA methylation levels of PDZK1 in glioma.** (**A**) Summary of the alteration rates of PDZK1 in glioma (cBioPortal). (**B**, **C**) DNA methylation changes in PDZK1 in glioma assessed using the UALCAN database. * p < 0.05 compared with the control. (**D**) The methylation levels of the PDZK1 CpG island were analyzed by utilizing the EWAS Data Hub. (**E**) The relationship between the methylation levels of PDZK1 CpG islands and the expression of PDZK1 mRNA was analyzed by utilizing the EWAS Data Hub. (**F**) The OS of patients with gliomas stratified by the methylation level of the PDZK1 CpG island was analyzed via the EWAS Data Hub.

The TCGA and cBioPortal databases were utilized to determine the genomic alteration profiles of PDZK1. As exhibited in Figure 2. Only 0.3% of the 1396 patients with gliomas presented with PDZK1 gene alterations (Figure 2A). Therefore, we next determined the methylation levels of the PDZK1 promoter in glioma by using the UALCAN database. Consistently, PDZK1 was more highly expressed in glioma tissues than in normal tissues (Figure 2B). The promoter methylation levels of PDZK1 were lower in GBM specimens than in normal tissue specimens (Figure 2C), indicating that promoter DNA hypomethylation may be the reason for the high expression of PDZK1 in glioma. Next, by utilizing the EWAS Data Hub, we found that the probe “cg05803361”, located at chr1 145727320, was related to the PDZK1 CpG island. The methylation levels of the PDZK1 CpG island were also lower in glioma tissues than in normal brain tissues (Figure 2D). Additionally, the PDZK1 mRNA expression level was negatively correlated with the methylation level of the PDZK1 promoter (Figure 2E). We further analyzed the prognostic value of the PDZK1 promoter DNA methylation level by using the EWAS Data Hub. The results showed that a low promoter DNA methylation level of PDZK1 was correlated with poor overall survival in patients with gliomas (Figure 2F). In conclusion, our findings indicate that promoter hypomethylation of the PDZK1 promoter was the reason for the high expression of PDZK1 in glioma, and the methylation level of PDZK1 is a meaningful prognostic marker in glioma.

### GO and KEGG enrichment analysis of proteins that potentially interact with PDZK1

**Figure 3:**
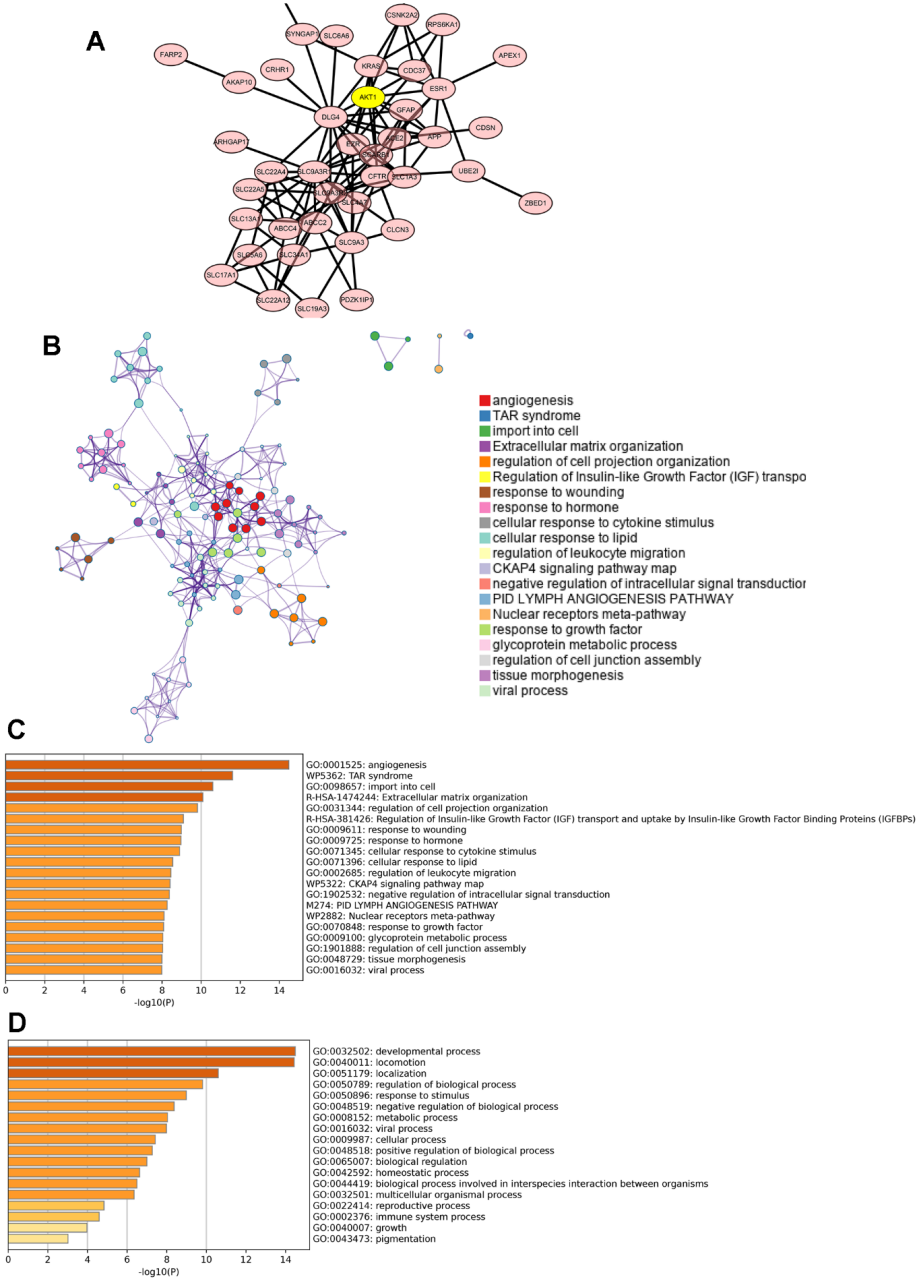
**Predicted functions and pathways of PDZK1-associated coexpressed genes in glioma.** (**A**) The PPI network was generated from the PDZK1-associated coexpressed genes, which were constructed using the Cytoscape database. (**B**–**D**) GO functional enrichment analysis and KEGG pathway analysis of PDZK1-associated coexpressed genes were conducted using the Metascape database.

We next constructed a PPI network by using the top 62 genes, which were obtained from the UALCAN database and BioGRID database, that were coexpressed and potentially interacted with PDZK1. AKT1, DLG4, SLC9A3R1 and ESR1 were most likely to interact with PDZK1 (Figure 3A). In addition, these genes were used to further determine the possible role of PDZK1 in glioma cells. Using the Metascape database, we performed GO term and KEGG pathway analyses. KEGG pathway analysis revealed that these genes were primarily associated with angiogenesis, TAR syndrome, TAR import into cells, extracellular matrix organization and regulation of cell projection organization (Figure 3B, 3C). The top-ranked Gene Ontology biological processes were developmental process, locomotion, localization and regulation of biological process (Figure 3D).

### PDZK1 knockdown impairs glioma cell proliferation and invasion

**Figure 4:**
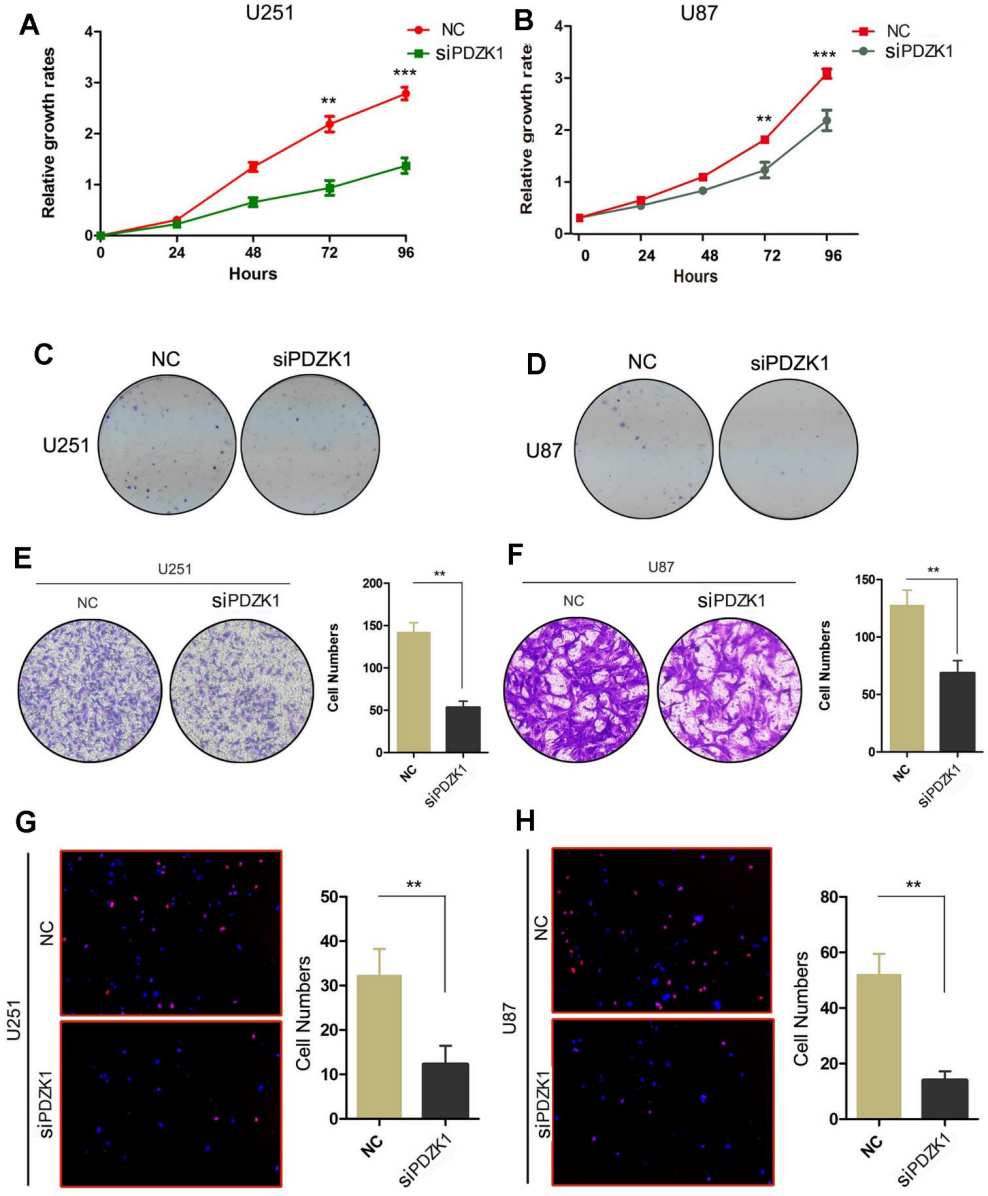
**PDZK1 knockdown inhibits the proliferation and invasion of glioma cells.** (**A**, **B**) The effect of PDZK1 knockdown on U251 and U87 cell proliferation was assessed by the CCK-8 cell growth assay. ***P < 0.001. (**C**, **D**) The effect of PDZK1 knockdown on U251 and U87 cell proliferation was assessed by a colony formation assay. (**E**, **F**) Transwell assays were used to detect the inhibitory effect of PDZK1 knockdown on invasion in U251 and U87 cells. **P < 0.01. (**G**, **H**) Knockdown of PDZK1 inhibited DNA replication in U251 and U87 cells, as determined by EdU staining. **P < 0.01.

Based on the above results, PDZK1 may act as an oncogene in glioma cells. Therefore, we investigated the effects of PDZK1 on the proliferation ability and colony formation ability of glioma cells. By conducting CCK-8 and colony formation assays, we found that compared with control cells, cells transfected with PDZK1 siRNA exhibited decreased proliferation and clonogenicity (Figure 4A–4D). Glioma cells frequently invade adjacent brain areas surrounding carcinomas *in situ*. We then conducted a Transwell assay to evaluate the effects of PDZK1 on glioma cell invasion ability. We observed that silencing PDZK1 expression through siRNA transfection decreased the number of cells transferred from the upper chamber (Figure 4E, 4F). Furthermore, the EdU assay was also used to measure the DNA replication ability of the cells, and the results showed that when PDZK1 was knocked down, the number of cells stained with EdU dramatically decreased (Figure 4G, 4H). In conclusion, our results demonstrated that knockdown of PDZK1 significantly inhibits glioma cell proliferation and invasion, indicating that PDZK1 may play a critical role in gliomagenesis.

### PDZK1 binds to AKT1 and maintains its phosphorylation

**Figure 5:**
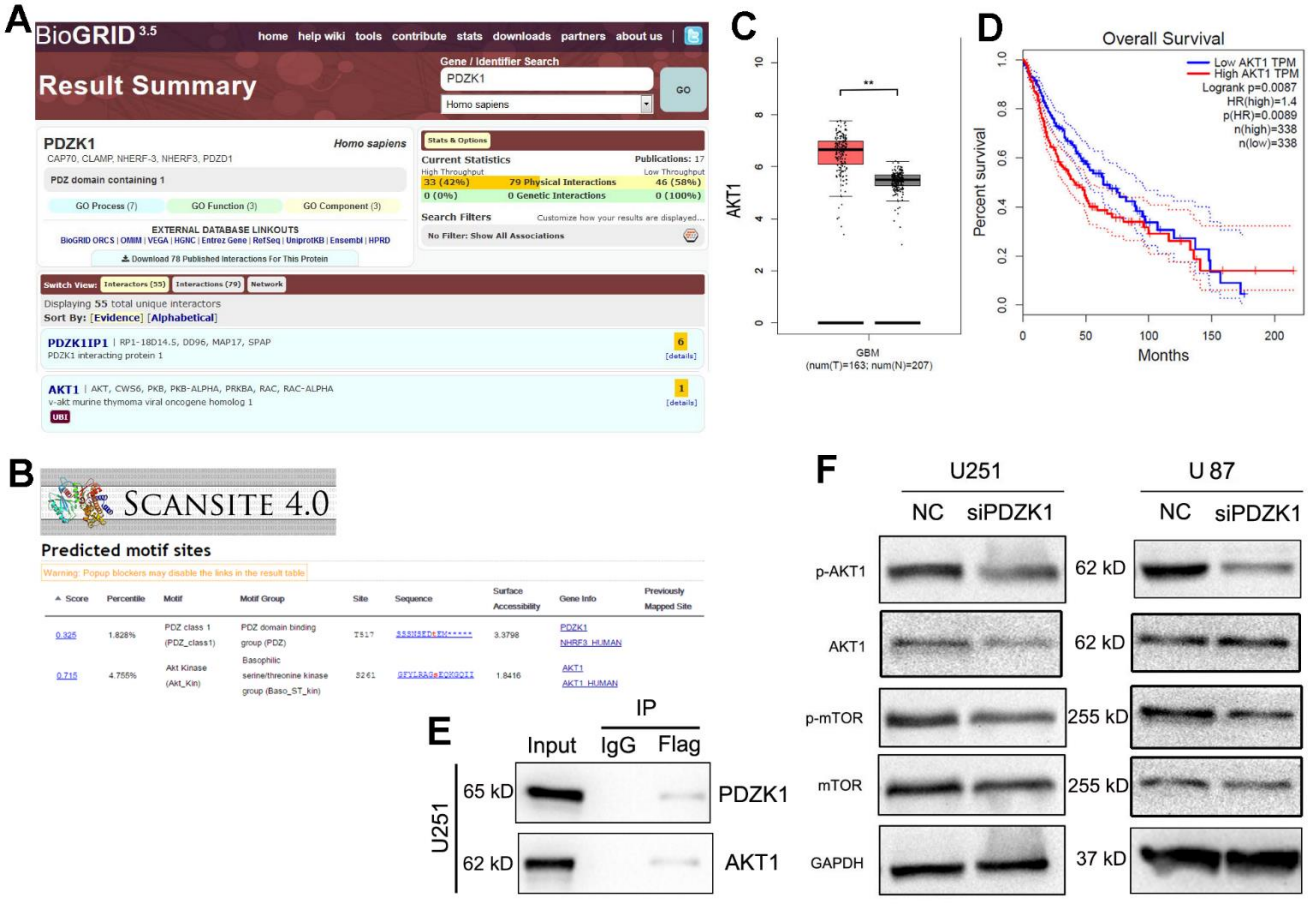
**PDZK1 binds to AKT1 and maintains its phosphorylation.** (**A**) BioGRID (version 3.5) software was used to screen for potential proteins that interact with PDZK1, and AKT1 was found to be a possible binding partner of PDZK1. (**B**) The “Scansite” (version 4.0) website was utilized to screen for potential proteins that interact with PDZK1, and AKT1 was also found to be a possible binding partner of PDZK1. (**C**) AKT1 expression data were obtained from the GEPIA datasets; GBM tissues had higher AKT1 expression levels than normal brain tissues. (**D**) Glioma patients with high AKT1 expression had poor survival. The Kaplan–Meier method was used for this analysis. (**E**) U251 cells were transfected with Flag-PDZK1. Coimmunoprecipitation showed the interaction between PDZK1 and endogenous AKT1 in U251 cells. (**F**) The protein expression of AKT1, p-AKT1, mTOR, p-mTOR and GAPDH was detected by Western blotting. PDZK1 knockdown significantly decreased the levels of phosphorylated AKT1 and mTOR proteins.

To explore the molecular function of PDZK1 in glioma cells, the “BioGRID” website was utilized to search for potential proteins that bind to PDZK1. AKT1 was found to be a possible binding partner of PDZK1 (Figure 5A). For more accurate and reliable predictions, another protein prediction website (ScanSite 4.0) was utilized; AKT1 was also present on the search results page (Figure 5B). Therefore, we selected AKT1 for further research. Most studies have revealed that aberrant AKT1 activation causes a wide variety of disorders, including various types of cancers. First, to confirm the role of AKT1 in glioma, we analyzed TCGA data and found that AKT1 was overexpressed in glioma tissues (Figure 5C). Consistently, a low level of AKT1 mRNA predicted poor prognosis in glioma patients (Figure 5D). To determine whether PDZK1 could bind to AKT1, we constructed a Flag-tagged PDZK1 expression vector and transfected this vector into U251 cells. Then, a coimmunoprecipitation experiment was conducted. The results showed that endogenous AKT1 coimmunoprecipitated with PDZK1 (Figure 5E). Protein-protein interactions, such as protein stability [19, 20], protein location, and protein modification [21], mediate multifaceted alterations in protein function. Next, we wanted to determine whether the binding of PDZK1 to AKT1 could alter the expression or phosphorylation of the AKT1 protein. Western blotting was used to investigate the effect of PDZK1 knockdown on the AKT1/mTOR signaling pathway in glioma cells. The results revealed that knockdown of PDZK1 inhibited the phosphorylation of AKT1/mTOR in U251 and U87 cells (Figure 5F) but had a small influence on the protein levels of AKT1 and mTOR. Taken together, our results revealed that PDZK1 interacted with AKT1 and that knockdown of PDZK1 may inhibit the activation of the AKT-mTOR signaling pathway in glioma cells.

### Knocking down PDZK1 induces glioma cell cycle arrest and cell apoptosis

**Figure 6:**
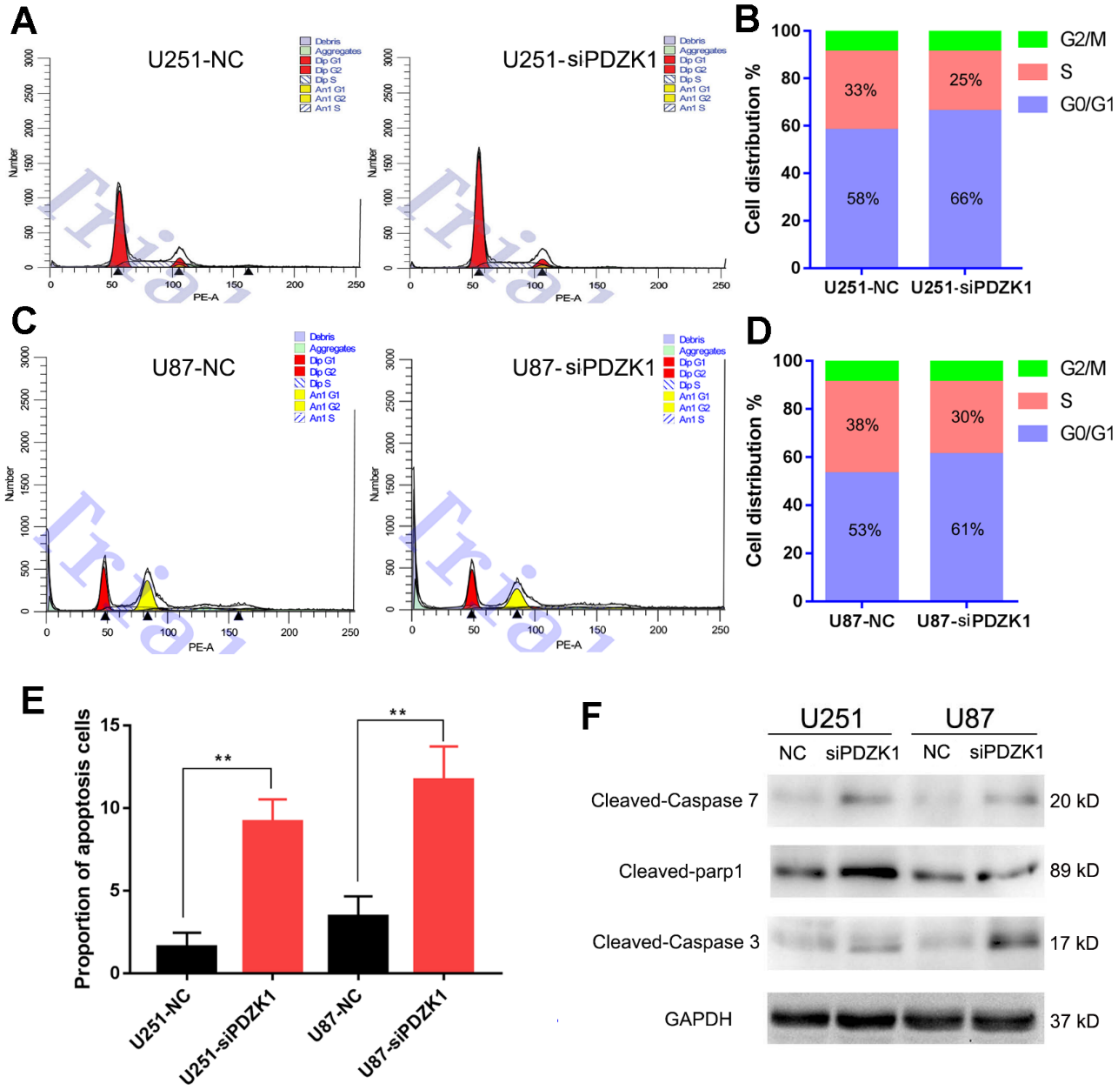
**Knockdown of PDZK1 induces glioma cell cycle arrest and cell apoptosis.** (**A**, **B**) PDZK1 knockdown induced cell cycle arrest in the G0/G1 phase. After transfecting U251 cells with PDZK1 siRNA for 24 h, the cells were collected for cell cycle analysis by PI staining. (**B**) Quantitation of cell cycle arrest in the G0/G1 phase induced by PDZK1 in (**B**). (**C**, **D**) PDZK1 knockdown induced cell cycle arrest in the G0/G1 phase. After transfecting U87 cells with PDZK1 siRNA for 24 h, the cells were collected for cell cycle analysis by PI staining. (**D**) Quantitation of cell cycle arrest in the G0/G1 phase induced by PDZK1 in (**D**). (**E**) The proportion of apoptotic cells was detected by flow cytometry. PDZK1 knockdown induced U251 and U87 cell apoptosis. (**F**) The protein expression of cleaved caspase-7, parp1, caspase-3 and GAPDH was detected by Western blotting. PDZK1 knockdown increased the expression of cleaved caspase-7, parp1, and caspase-3.

AKT is a critical mediator of cell growth and apoptosis, and abnormally activated AKT kinase is closely related to carcinogenesis. To determine whether PDZK1 can mediate the cell cycle in glioma cells by interacting with AKT1, we used flow cytometry to investigate the effects of PDZK1 on cell cycle progression. When the expression of PDZK1 was suppressed in glioma cells, the percentage of U251 cells in the G0/G1 phase increased from 58% to 66%, whereas the percentage of cells in the S phase decreased from 33% to 25% (Figure 6A, 6B). Similarly, siRNA-mediated knockdown of PDZK1 in U87 cells increased the percentage of cells in the G0/G1 phase from 53% to 61%, whereas the percentage of cells in the S phase decreased from 38% to 30% (Figure 6C, 6D). In addition, we also conducted flow cytometry to examine cell apoptosis and found that the percentage of apoptotic cells was significantly increased after silencing PDZK1 in both U251 and U87 cells (Figure 6E). Western blotting also showed that PDZK1 knockdown upregulated the levels of activated caspase 3, caspase 7 and PARP proteins in U251 and U87 cells (Figure 6F). Thus, the above results indicated that knockdown of PDZK1 led to cell cycle arrest at the G1/S checkpoint and increased cell apoptosis in glioma cells.

## DISCUSSION

Glioma is the most common tumor of the central nervous system [22–24], and gliomas are classified as WHO grades I–IV based on tissue classification and staging [25]. In recent decades, despite rapid improvements in the treatment of glioma, the prognosis for glioma patients has remained poor [26]. Understanding the mechanism of glioma progression (aberrant oncogene activation or tumor suppressor gene inactivation) could provide a theoretical basis for glioma treatment.

PDZK1 is a scaffolding protein containing a PDZ domain located on chromosome 1 [27]. Previous studies have reported that PDZK1 inhibits RCC malignant manifestations by suppressing the phosphorylation of SHP-1 and subsequently reducing the phosphorylation of AKT1 [18]. Zheng also showed that downregulation of PDZK1 predicts poor prognosis in RCC patients. Pei Yu reported that PDZK1 in white blood cells is resistant to apoptosis and necrotic core development in atherosclerotic plaques [28]. Another study reported that PDZK1 could mediate the activation of urate-anion exchangers, thereby promoting the ability to reabsorb uric acid [29]. In addition, Shimizu demonstrated that PDZK1 physically binds with ABCG2 and regulates breast cancer resistance [30]. Zhao et al. reported that PDZK1 could modify the phosphorylation of PTEN and promote the PI3K/AKT signaling pathway in gastric cancer. Notably, the role of PDZK1 in glioma is still not defined. By using the TCGA and CGGA glioma databases, we found that PDZK1 is expressed at high levels in glioma tissues. This finding indicated that PDZK1 may be a potential oncogene for glioma. Our further studies revealed that knockdown of PDZK1 in glioma cells could inhibit glioma cell proliferation, colony formation and invasion, which confirmed that PDZK1 could be a cancer-promoting gene for glioma.

We also explored the mechanism by which PDZK1 suppresses glioma cell proliferation. First, we used the “BioGRID” website to search for possible proteins that bind to PDZK1. AKT1 was found to be a binding partner of PDZK1. Coimmunoprecipitation confirmed that PDZK1 interacts with AKT1 in glioma cells. Studies have reported that PDZK1 reduces AKT1 phosphorylation and inhibits RCC cell proliferation [18]. In our study, we found that PDZK1 formed a complex with AKT1 in glioma, suggesting that PDZK1 could directly affect the function of AKT1. Robust AKT activation, which promotes tumor aggressiveness, has been widely acknowledged [31, 32]. We also confirmed the role of AKT1 in glioma by analyzing TCGA data and found that AKT1 was overexpressed in glioma specimens. Consistently, a low level of AKT1 mRNA predicted poor prognosis in glioma patients, indicating that AKT1 plays a tumorigenic role in glioma. Moreover, western blotting further revealed that PDZK1 knockdown also reduced the activation of AKT1 (phosphorylated AKT1). It has been reported that AKT1 promoted Mdm2 entry into the nucleus, thereby accelerating the degradation of the p53 protein [33]. PDZK1 knockdown suppressed the phosphorylation of AKT1, and the inhibition of AKT1 maintained the stability of the p53 protein, which then resulted in cell cycle arrest. This may be the mechanism by which PDZK1 acts as a tumorigenic gene for glioma. All our results preliminarily confirmed that the loss of PDZK1 expression by siRNA inhibits glioma cell proliferation and induces cell apoptosis by preventing AKT1 phosphorylation. However, the present study also has limitations, such as how PDZK1 mediates AKT1 phosphorylation and why PDZK1 knockdown decreases phosphorylated AKT1 levels.

Overall, this research illustrated that PDZK1 is overexpressed in glioma specimens. Upregulated PDZK1 was demonstrated to be correlated with adverse prognosis in glioma patients. Knockdown of PDZK1 delayed cell growth, inhibited the cell cycle, and mediated apoptosis in glioma cells. PDZK1 promotes tumor development by regulating the phosphorylation of AKT1 (Figure 5). Hence, our data indicate that PDZK1 acts as a tumorigenic gene in glioma by maintaining the activation of the AKT/mTOR signaling pathway. Moreover, our data also indicated that PDZK1 may be a potential therapeutic target for glioma.

## Supplementary Material

Supplementary Figure 1
