# Supplementary material for: Promoter hypomethylated PDZK1 acts as a tumorigenic gene in glioma by interacting with AKT1
Source: Aging (Albany NY). 2024 Apr 25;16(8):7174–87. doi: 10.18632/aging.205750 (PMC11087087; doi:10.18632/aging.205750)
Supplement: Supplementary Figure 1 [file aging-16-205750-s001.pdf]

SUPPLEMENTARY FIGURE

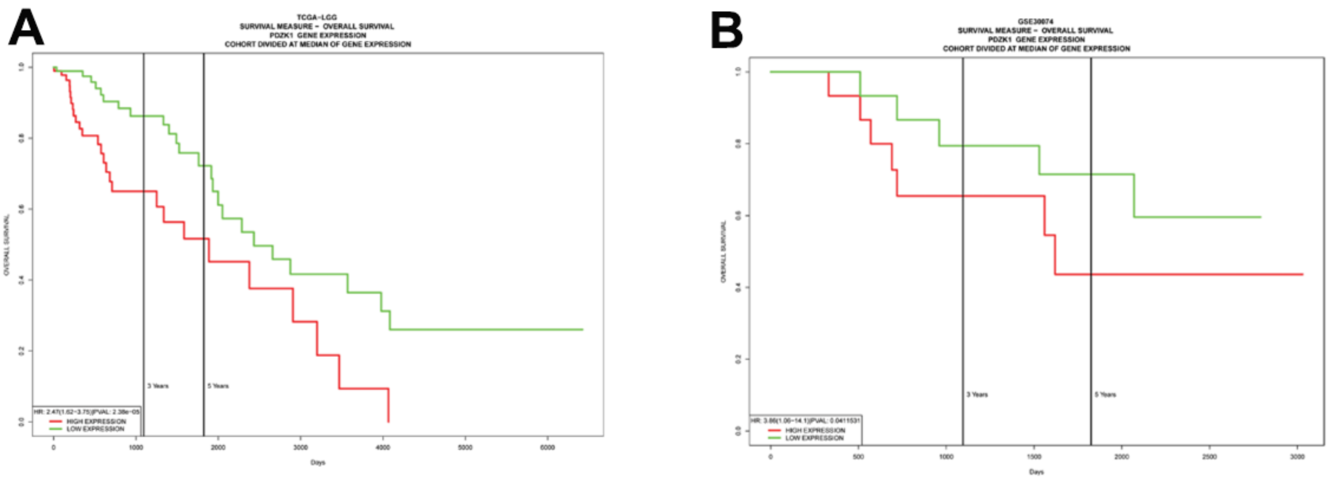

Supplementary Figure 1. High PDZK1 expression is associated with poor prognosis. The glioma patients with a lower PDZK1 expression had a more favourable survival time than glioma patients with a higher PDZK1 expression. The Kaplan–Meier method was used for this analysis. (**A**) Data from TCGA-LGG; (**B**) data from GSE30074 dataset.
